# Supplementary material for: Identification Mechanism of BACE1 on Inhibitors Probed by Using Multiple Separate Molecular Dynamics Simulations and Comparative Calculations of Binding Free Energies
Source: Molecules. 2023 Jun 15;28(12):4773. doi: 10.3390/molecules28124773 (PMC10305653; doi:10.3390/molecules28124773)
Supplement: Supplementary file 1 [file molecules-28-04773-s001.zip › molecules-2451708-supplementary.pdf]

Identification Mechanism of BACE1 on Inhibitors Probed by Using Multiple Separate Molecular Dynamics Simulations and Comparative Calculations of Binding Free Energies

Yiwen Wang[1,2], Fen Yang[1], Dongliang Yan[1,3], Yalin Zeng[1], Benzheng Wei[4], Jianzhong Chen[1,3,*] and Weikai He[1,2,*]

[1] School of Information Science and Electrical Engineering, Shandong Jiaotong University, Jinan 250357, China; 15550053180@163.com (Y.W.); fen_yang0223@163.com (F.Y.); 15367294293@163.com (Y.Z.)
[2] School of Aeronautics, Shandong Jiaotong University, Jinan 250357, China
[3] School of Science, Shandong Jiaotong University, Jinan 250357, China
[4] Center for Medical Artificial Intelligence, Shandong University of Traditional Chinese Medicine, Qingdao 266112, China; wbz99@sina.com
* Correspondence: jzchen@sdjtu.edu.cn (J.C.); 214035@sdjtu.edu.cn (W.H.)

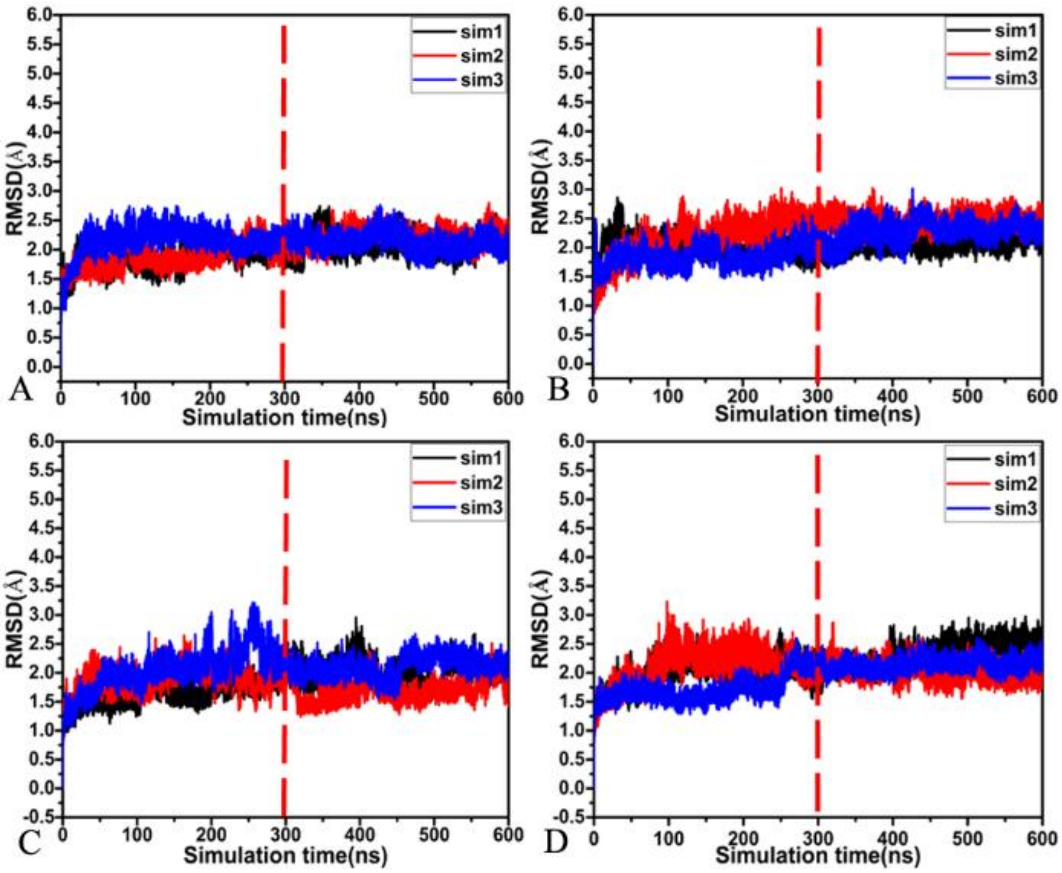

Figure S1. RMSDs of backbone atoms in BACE1 calculated by using three separate MD trajectories: (A) the *apo* BACE1, (B) the 60W-BACE1 complex, (C) the 954-BACE1 complex and (D) the 60X-BACE1 complex.

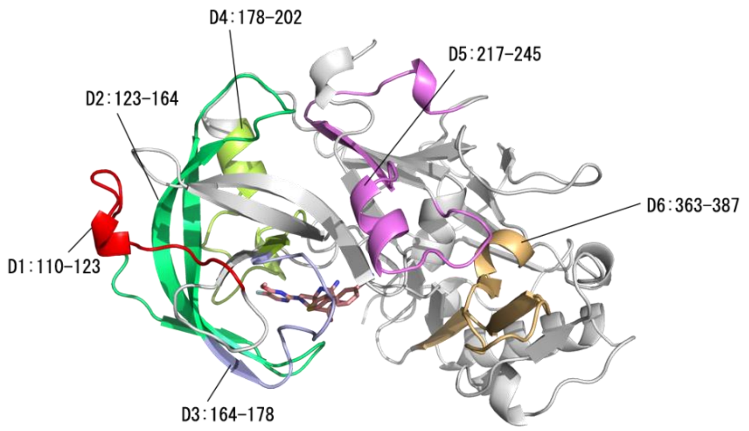

Figure S2. Structural domains corresponding to the obvious changes in RMSFs due to inhibitor binding.

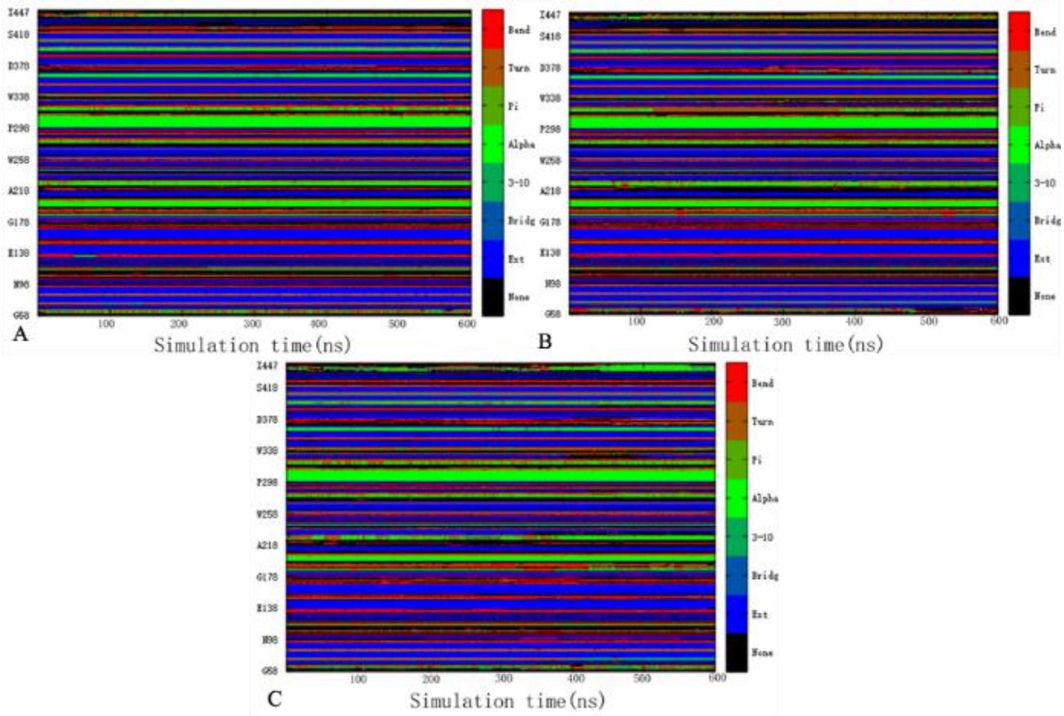

Figure S3. Stability of secondary structures for the 60W-bound BACE1 in three separate MD simulations: (A) the simulation 1, (B) the simulation 2 and (C) the simulation 3.

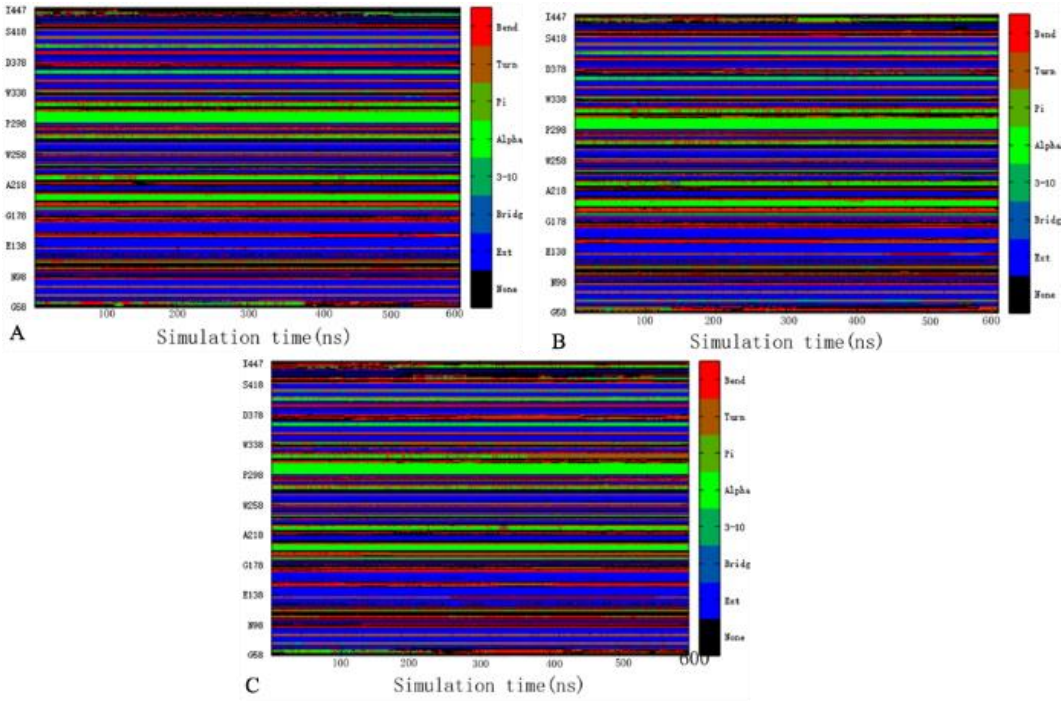

Figure S4. Stability of secondary structures for the 954-bound BACE1 in three separate MD simulations: (A) the simulation 1, (B) the simulation 2 and (C) the simulation 3.

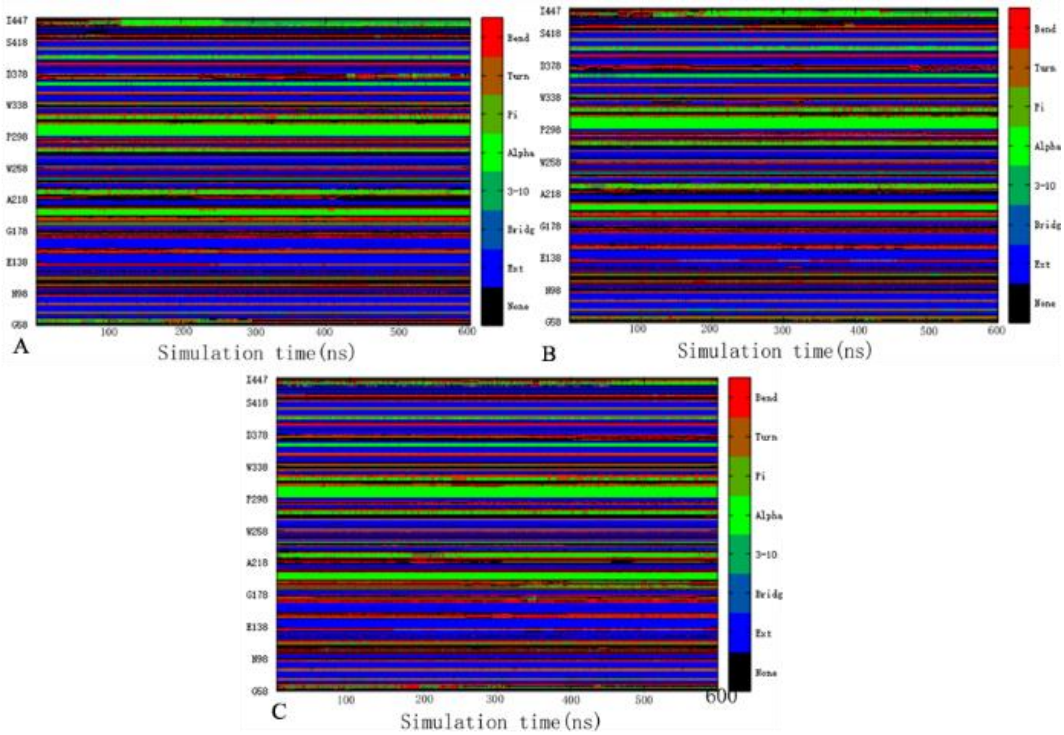

Figure S5. Stability of secondary structures for the 60X-bound BACE1 in three separate MD simulations: (A) the simulation 1, (B) the simulation 2 and (C) the simulation 3.

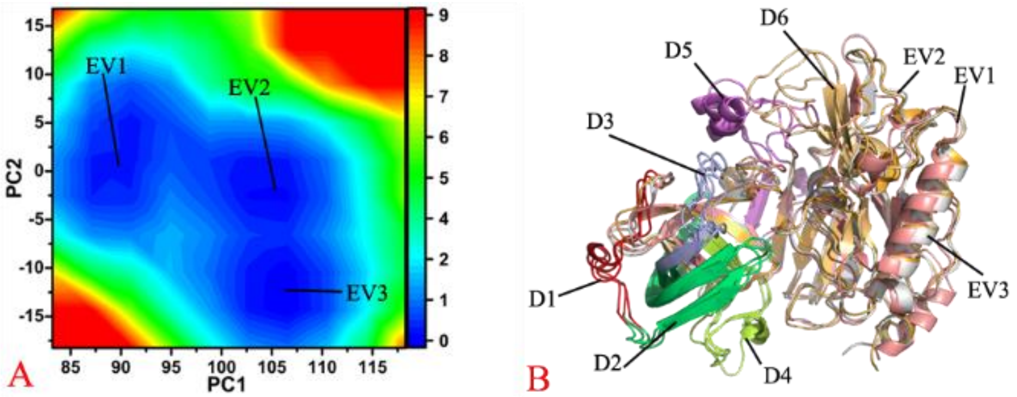

Figure S6. (A) Free energy landscapes of the *apo* BACE1 constructed by using the PC1 and PC2 as reaction coordinates and (B) structural superimposition of the *apo* BACE1 situated at the energy valleys EV1-EV3.
